# Newborn and family-centered care: a scoping review on related newborn rights and principles

**DOI:** 10.7189/jogh.16.04226

**Published:** 2026-06-26

**Authors:** Andrea Togo, Ornella Lincetto, Jenny Bua, Ilaria Mariani, Marzia Lazzerini

**Affiliations:** 1 Institute for Maternal and Child Health, IRCCS “Burlo Garofolo”, WHO Collaborating Centre for Maternal and Child Health, Trieste, Italy; 2 Neonatal Intensive Care Unit, Institute for Maternal and Child Health, IRCCS “Burlo Garofolo”, Trieste, Italy

## Abstract

**Background:**

Newborn- and family-centered care (N&FCC) is essential for improving newborn and parental outcomes, yet no previous review focused on related newborn rights and principles. In this scoping review, we aimed to identify and synthetise related rights of hospitalised newborns and principles of N&FCC enunciated in the literature, assess correspondence of rights with international human rights instruments, examine alignments between rights and principles, and identify gaps.

**Methods:**

We searched PubMed, EMBASE, Web of Science, and Google Scholar up to 20 August 2025 for records that explicitly described the rights of hospitalised newborns or articulated principles relevant to N&FCC. We thematically synthesised the identified rights and principles into overarching sets, assessed them for mutual alignment, and mapped rights to five fundamental international human rights instruments.

**Results:**

Twelve of the 13 447 retrieved records were eligible for inclusion. We identified 40 enunciated rights and 57 principles, with no single source providing a comprehensive list of either. Thematic analysis yielded 12 overarching sets of rights and 6 overarching sets of principles which showed substantial mutual alignment. Each set of rights was supported by two or more international human rights instruments. The analysis of principles enunciated so far in the literature highlighted that newborns are not always explicitly identified as the intended subject of N&FCC. Newborn rights and N&FCC principles were predominantly developed by groups/initiatives in high income countries.

**Conclusions:**

This review provides a comprehensive synthesis of newborn rights and N&FCC principles, demonstrating correspondence between newborn rights and international human rights law instruments, and alignment between rights and principles, thereby strengthening conceptual foundations of N&FCC. Our findings suggest that N&FCC should be considered a rights-based entitlement of hospitalised newborns, rather than solely a model of care. Future work should clarify the intended focus of N&FCC, examine mechanism for translating newborn rights into standards of care, and involve stakeholders from both high- and low- and middle-income countries to support its wider implementation.

Over thirty million newborns need hospital care each year at different levels, ranging from short stays in basic neonatal units to prolonged hospitalisations in neonatal intensive care units (NICUs) for complex or life-threatening conditions [1]. Early-life environmental factors can influence infants’ developmental trajectories [2]. This is particularly relevant for newborns who are born small, preterm, or unwell and require hospitalisation. Although hospital neonatal care is often life-saving and essential for reducing long-term morbidity, it may also introduce separation from family, reduced opportunities for early parent-infant bonding, exposure to painful or invasive procedures, disruption of sensory regulation [3–7]. These exposures may affect not only the newborn’s developmental trajectory, but also its family’s emotional well-being and the establishment of early parent–infant relationships [8,9]. In the context of hospitalised newborns, the provision of both newborn-centered care and family-centred care (N&FCC), *i.e.* the support of newborns’ unique individual needs and the enabling of an active role of the family in close collaboration with the healthcare workers, respectively, are increasingly recognised as essential for improving the experience of care and, ultimately, health outcomes [10–12]. This approach should be understood within a continuum of care that extends beyond hospitalisation and is sustained after discharge, in the home and community, beyond the neonatal period. It is also consistent with the Nurturing Care Framework, which emphasises good health, adequate nutrition, safety and security, responsive caregiving, and opportunities for early learning throughout early childhood [13].

In neonatal inpatient settings, experience of care is a core component of quality of care in health systems and is closely linked to the respect, protection, and fulfilment of newborns’ rights, as reflected in the World Health Organization (WHO) framework for improving the quality of care for small and sick newborns [14]. Despite major advances in human rights frameworks for maternal and child health in hospital settings, such as the Respectful Maternity Care Charter [15], the specific rights of small and sick newborns during hospitalisation have not been articulated or synthesised within a single document [16–19]. From birth, irrespective of hospitalisation status, the newborn is considered an individual with rights, as well as specific communicative capacities and developmental needs [20,21].

For small and sick newborns requiring hospital care, N&FCC has been shown to support care tailored to the infant’s health status, cues, and developmental needs [21]. It can also empower parents in partnership with health professionals, strengthen their caregiving skills, support communication and bonding, and ultimately promote the well-being of both newborns and families [22–25]. However, the relationship between N&FCC and newborn rights has not previously been examined systematically.

Notwithstanding increasing recognition of the significance of N&FCC by multiple institutions, including the WHO and scientific societies [1,14,26–29], implementation remains uneven across and within geographical regions [16]. This may partly be reflective of the substantial heterogeneity in N&FCC definitions and models of care, which itself is likely to arise from multiple factors, including the broad and multidimensional nature of N&FCC interventions and the need to adapt them to different settings, shaped by sociocultural norms, values and beliefs, as well as by resource availability, service organisation, and workforce capacity [22,30,31]. A recent systematic review of 91 records identified 40 different definitions of N&FCC and 28 different models of care proposed over time and across settings, with marked variation in the terminology used to describe similar models of care [22]. Among these models, 51 categories of interventions were identified and summarised into five macro categories. Two other systematic reviews of randomised controlled trials similarly highlighted major heterogeneity among interventions for implementing N&FCC, with a lack of head-to-head comparisons hampering the assessment of relative effectiveness [31,32]. Although this heterogeneity may, therefore, partly reflect the broad and context-sensitive nature of N&FCC, it may also pose important challenges for policymakers by limiting the availability of robust evidence to guide decision-making, hindering the development of reliable standards and indicators, restricting comparison across settings, and contributing to inconsistent implementation of N&FCC globally. The variability in N&FCC implementation in hospital settings may also reflect the absence of clearly defined and universally accepted underpinning principles. This lack of conceptual clarity can contribute to inconsistent interpretation and operationalisation across settings [30].

For this review, we defined rights and principles as follows: rights articulate moral and legal entitlements to have or be able to do something, and can be understood as the ‘what’ that should be done [33], while principles are fundamental truths or propositions that serve as the foundation for a system of belief or behaviour, or for a chain of reasoning, and explain the ‘why’ behind actions [34].

To date, no systematic review has comprehensively analysed the rights of hospitalised newborns alongside principles of N&FCC for inpatient settings, nor systematically investigated their relationships with key international human rights law instruments. Such a systematic identification of the rights of hospitalised newborns and N&FCC principles enunciated in existing literature, including grey literature, together with the examination of their interrelationship and correspondence with international human rights instruments, is essential for strengthening both the legal grounding and the conceptual foundation for N&FCC, thereby favouring a coherent human rights approach to newborn health [35].

To address this gap, we conducted a scoping review with two key objectives **(****Figure 1**):

**Figure 1:**
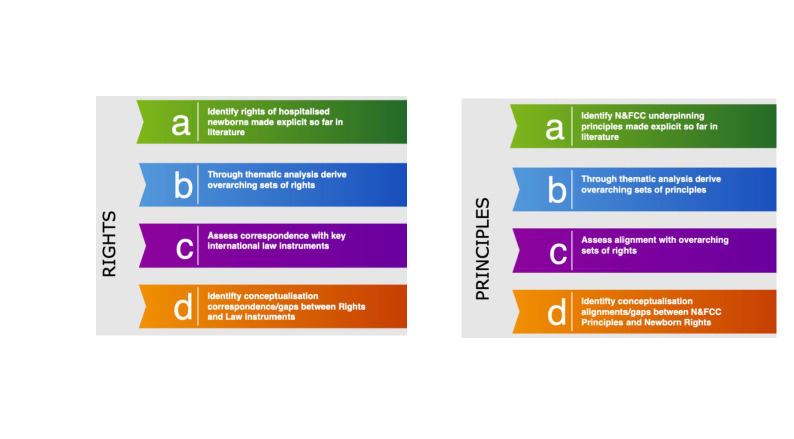
Objectives and key steps in the study methodology.

– In relation to rights, to identify the rights of hospitalised newborns as reported in the literature; compare them through thematic analysis and derive overarching sets of rights; assess their correspondence with key international human rights law instruments as ‘gold standard’; and identify correspondence or gaps in conceptualisation.

– In relation to principles, to identify the underpinning principles of N&FCC in hospital settings as reported in the literature; compare them through thematic analysis and derive overarching sets of principles; assess their alignment with the above-mentioned overarching sets of newborn rights; and identify alignments or gaps in conceptualisation.

## METHODS

### Study design

This scoping review followed the Joanna Briggs Institute guidelines and Arksey’s framework for scoping reviews, including its most recent updates [36–38]. We developed a review protocol prior to commencing the screening process, but did not register it with PROSPERO, as it does not accept protocols for scoping reviews. We followed the PRISMA-ScR in reporting our findings (Table S1 in the **Online Supplementary Document**) [39,40].

### Search strategy

We searched PubMed, EMBASE, Web of Science, and Google Scholar for records published up to 20 August 2025. We included Google Scholar because it provides access to a broad and comprehensive range of records on patient rights, including grey literature [41,42]. To develop the search strategy (Table S2 in the **Online Supplementary Document**), we first tabulated and compared the keywords used in previous studies [31,32]. We then refined the strategy by incorporating additional keywords identified from an initial set of retrieved studies and by optimising the use of Boolean operators, after which we tested it to ensure that it successfully retrieved all relevant studies, including those reported in prior reviews [31,32]. In addition, for both objectives, we manually screened the reference lists of included studies with no restrictions in terms of the language or publication date applied. We imported all records into EndNote, version 21.5 (Clarivate, London, UK) and removed any duplicates.

### Inclusion and exclusion criteria

Records were eligible if they explicitly articulated the rights of newborns within inpatient settings and/or if they defined and/or referred to principles of N&FCC in hospital settings.

We also selected the most recent document on patient rights from the WHO Patient Safety Rights Charter, given the applicability of its provisions to newborns in inpatient care [43]. We excluded records that did not explicitly reference newborns or neonatal care in inpatient settings or that did not provide extractable content on rights and/or N&FCC principles (*e.g.* documents in which rights/principles were only implied or mentioned in passing), and those that were written in languages not spoken by the review team (*e.g.* Chinese).

### Record selection

Three authors (AT, OL, JB) screened a sample of titles/abstracts for 20 records to ensure the consistent interpretation and application of inclusion/exclusion criteria. Subsequently, one author (AT) screened all remaining titles and abstracts and retrieved the full texts of the relevant records. Then, to minimise selection bias, three authors (AT, OL, JB) independently reviewed the full texts of all records that satisfied the inclusion criteria at the title/abstract screening stage. Any discrepancies were resolved through discussion and consensus.

### Data extraction

We extracted data on the authors‘ names, issuing body of each included record (*e.g.* research groups, professional associations, international organisations, parent advocacy groups), year of publication, country affiliation of the first author, and the corresponding country income category (high-income countries (HICs) *vs*. low- and middle-income countries (LMICs), per the World Bank categorisation [44]) from each included record. For documents published by international organisations, no geographical classification was assigned.

### Data analysis

We first analysed all included records and extracted individual rights and principles, tabulating and preparing them for analysis, after which we performed a two-stage thematic analysis in line with thematic analysis methods [45]. Three authors (AT, OL, JB) independently conducted the data analysis through a multi-step process, followed by iterative comparison of interpretations and consensus-based resolution of discrepancies with all authors to reduce potential bias.

We carried out the analysis for objective 1 in four consecutive steps (**Figure 1**). In the first stage, three authors (AT, OL, JB) conducted open coding for each record to identify its core conceptual meaning. Initial codes were generated inductively from the content of each source, with attention to both explicit terminology and underlying conceptual intent. We conducted this coding iteratively, reviewing and redefining codes through discussion to ensure consistency of interpretation. Because the aim of the analysis was conceptual synthesis, we did not consider a formal calculation of inter-coder reliability to be appropriate; instead, we resolved discrepancies in coding and category assignment through discussion and consensus. We executed this process manually in Microsoft Excel, rather than in a dedicated qualitative analysis software, as the volume and nature of the data were considered amenable to manual management. In the second stage, we gropued codes using an axial coding framework to derive overarching sets of rights, defined as broad, higher-level rights that encompass and connect more specific newborn rights. Overarching categories were developed on the basis of conceptual similarity between codes assigned to each category, internal coherence within each category, and clear conceptual distinction between categories. This process allowed heterogeneous concepts to be consolidated into coherent units suitable for subsequent comparative analysis [46,47]. In the third stage, from the human rights treaty body system, we selected the five key international human rights instruments for their relevance and applicability to the rights of hospitalised newborns: the Universal Declaration of Human Rights (UDHR), which carries significant moral authority, and four core international human rights treaties; the Convention on the Rights of the Child (CRC); the International Covenant on Civil and Political Rights (ICCPR); the International Covenant on Economic, Social and Cultural Rights (ICESCR), and the Convention on the Rights of Persons with Disabilities (CRPD). The last four are legally binding for ratifying States and serve as the foundation for national human rights legislation, including those of children [48–50]. To assess correspondence with these instruments, we mapped each overarching right to relevant articles of the UDHR, CRC, ICCPR, ICESCR and CRPD. In the fourth stage, we identified areas of conceptual correspondence and gaps through analysis of the mapping.

We similarly carried out the analysis for objective 2 in four sequential stages (**Figure 1**). In the first stage, we open coded each extracted principle to identify its core conceptual meaning. Initial codes were generated inductively from the content of each source, with attention to both explicit terminology and underlying conceptual intent. Coding was undertaken as an iterative process, with codes reviewed and refined through discussion among the authors to ensure consistency in interpretation. As per objective one, we did not assess formal inter-coder reliability, but instead resolved discrepancies through discussion. Coding and synthesis were conducted as above. In the second stage, we grouped codes using an axial coding approach to derive overarching sets of principles, defined as broad categories that capture common elements across multiple principles. Overarching categories were developed on the same basis as for objective one. Third, we assessed the relationship between overarching sets of rights and overarching sets of principles through reciprocal mapping and the construction of an alignment matrix to capture many-to-many relationships. For this relationship analysis, we adopted the Cambridge Dictionary definition of ‘alignment’ as a state in which two entities are the same, similar, or in agreement, as opposed to being different or in conflict [51]. Here, alignment between overarching sets of rights and overarching sets of principles refers to the extent to which they are mutually consistent, coherent, and reinforcing, rather than contradictory. Fourth, we identified areas of conceptual alignment and gaps through analysis of the mapping, indicating where further clarification or development is required to strengthen the coherence between overarching rights and principles of N&FCC.

## RESULTS

The searches yielded a total of 13 447 records, with 13 201 retrieved from databases and an additional 246 from reference lists and website searches (**Figure 1**). After screening and exclusion of duplicates, we retrieved 19 records for eligibility assessment for eligibility. Following full text screening and authors discussion, we retained 12 records for analysis, relevant to either objective 1 (n = 4) or objective 2 (n = 8).

### Objective 1

#### Identified rights

Four of the 12 records met the inclusion and exclusion criteria for objective 1 [43,52–54]. Of these, two were issued by international organisations involving stakeholders from both HICs and LMICs [43,52], while the remaining two originated in HICs: one from a multidisciplinary research group [53] and one from a parent advocacy group [54]. Two records were published in the past two years, and the other two were published after 2010. Each included record articulated 10 rights, for a total of 40 identified rights (Table S3 in the **Online Supplementary Document**). No single record provided a comprehensive account of neonatal overarching sets of rights in hospital settings.

#### Overarching sets of rights for hospitalised newborns

**Figure 2:**
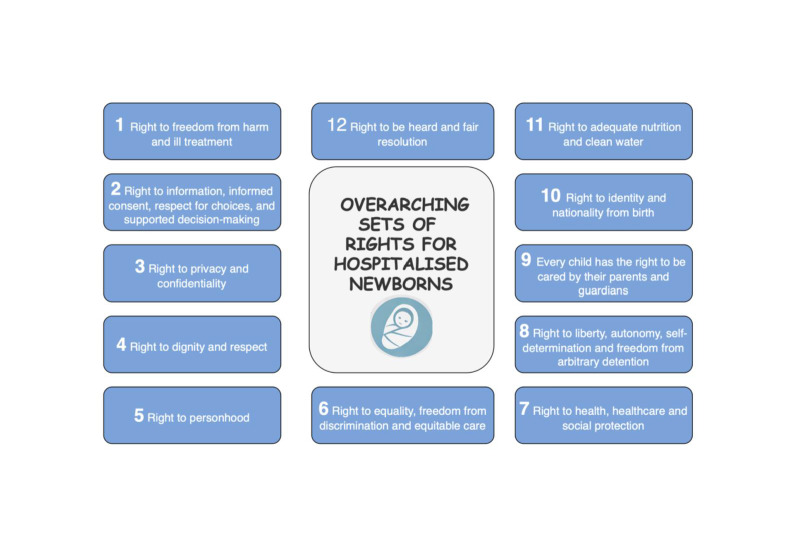
Overarching sets of rights for hospitalised newborns.

Thematic synthesis generated 12 overarching rights for hospitalised newborns, encompassing: freedom from harm and ill treatment; informed and family-supported decision-making; privacy; dignity and respect; personhood; equality and freedom from discrimination; health, healthcare and social protection; liberty and autonomy; parental presence and care; legal identity and nationality; adequate nutrition; and to be heard and fair resolution. **Box 1** and **Figure 2** detail the 12 overarching sets of rights for hospitalised newborns resulting from the thematic analysis of the 40 identified rights (Table S3 in the **Online Supplementary Document**).

Box 1 **Identified overarching sets of rights for hospitalised newborns**

**Right to freedom from harm and ill treatment**

Hospitalised newborns have the right to be free from harm and ill-treatment, including protection from unsafe, neglectful, or abusive practices in all aspects of clinical care.

**Right to information, informed consent, respect for choices and preferences, and supported decision-making**

Hospitalised newborns, through their parents or legal guardians, have the right to timely, accurate, and accessible information; access to medical records; supported decision-making; and to provide or withhold informed consent for all medical procedures, including those undertaken for research purposes, with respect for family choices and preferences.

**Right to privacy and confidentiality**

Hospitalised newborns have the right to privacy and confidentiality, including protection of their personal, medical, and family information in all aspects of care, documentation, and communication.

**Right to dignity and respect**

Hospitalised newborns have the right to be treated with dignity and respect in all aspects of care, irrespective of health status or prognosis, including in the provision of palliative care.

**Right to personhood**

Hospitalised newborns have the right to life and are recognised as persons and individual rights-holders from the moment of birth, not solely as patients or dependants, with their own identity, interests, and moral worth.

**Right to equality, freedom from discrimination and equitable care**

Hospitalised newborns have the right to equality, freedom from discrimination, and equitable access to quality care, irrespective of sex, disability, gestational age, ethnicity, socioeconomic status, or family circumstances.

**Right to health, healthcare, and social protection**

Hospitalised newborns have the right to the highest attainable standard of health, including timely, effective, evidence-based, and appropriate care delivered through safe healthcare processes by qualified and competent health workers, using safe medical products within secure healthcare facilities. This right includes access to neuroprotective and developmentally appropriate care, appropriate assistance during birth, care provided in the most suitable setting according to clinical needs, social protection and, where indicated, proportionate treatment, including palliative care, while avoiding both therapeutic obstinacy and euthanasia.

**Right to liberty, autonomy, self-determination and freedom from arbitrary detention**

Hospitalised newborns have the right to liberty and freedom from arbitrary detention, with any restriction of movement, separation or prolonged hospitalisation justified by clinical necessity, proportionate and consistent with the newborn’s best interests. Autonomy and self-determination are exercised through parents or legal guardians, who have the right and responsibility to participate meaningfully in decisions affecting the newborn’s care, subject to the child’s best interests and applicable legal safeguards.

**Every child has the right to be cared by their parents and guardians**

Hospitalised newborns have the right to be with and cared for by their parents or legal guardians, including guaranteed proximity, opportunities for bonding and meaningful family engagement in care. This right encompasses family-centered care that supports parental presence and participation, and recognises the importance of parents’ health and well-being in enabling optimal newborn care.

**Right to identity and nationality from birth**

Newborns are entitled to recognition of their legal identity from birth, including birth registration, name and nationality, irrespective of health status or place of birth.

**Right to adequate nutrition and clean water**

Hospitalised newborns have the right to safe, adequate, and appropriate nutrition and access to clean water, including support for breastfeeding or clinically indicated alternatives.

**Right to be heard and fair resolution**

The views and best interests of the newborn, as represented by parents or guardians, should be considered in care decisions, with access to advocacy and fair, transparent mechanisms for raising concerns or complaints and seeking resolution.

#### Correspondence between overarching sets of rights and international human rights instruments

The 12 overarching sets of rights mapped well to the international human rights instruments, *i.e. *the UDHR, CRC, ICCPR, ICESCR, and CRPD (**Table 1**). Speficially, each set of rights was supported by two or more international human rights instruments, indicating that the rights described in the medical literature for hospitalised newborns have strong normative correspondence with international human rights law.

**Table 1:** Overarching sets of rights and correspondence with International Human Rights Instruments

Overarching sets of rights for hospitalised newborns	UDHR	CRC	ICCPR	ICESCR	CRPD
Right to freedom from harm and ill-treatment	Art. 5	Art. 19	Art. 7	-	Art. 15, Art. 16
Right to information, informed consent, respect for choices and preferences, and supported decision-making	-	-	Art. 18, Art. 19	-	Art. 21
Right to privacy and confidentiality	Art. 12	Art. 16	Art. 17	-	Art. 22
Right to dignity and respect	Art. 1	-	-	-	Art. 3
Right to personhood	Art. 6	-	Art. 1, Art. 16	-	Art. 17
Right to equality, freedom from discrimination, and equitable care	Art. 2, Art. 7	Art. 2	Art. 2, Art. 3, Art. 26	Art. 2, Art. 3	Art. 5, Art. 7
Right to health, health care and social protection	Art. 25	Art. 3.3, Art. 24.1	-	Art. 9, Art. 10, Art. 12	Art. 25, Art. 28
Right to liberty, autonomy, self-determination, and freedom from arbitrary detention	Art. 9	-	Art. 9, Art. 19, Art. 24	Art. 1	Art. 3
Every child has the right to be with their parents or guardians	-	Art. 9	Art. 23	-	Art. 23
Right to an identity and nationality from birth	-	Art. 7	Art. 24	-	Art. 18
Right to adequate nutrition and clean water	Art. 25	Art. 24	-	-	Art. 28
Right to be heard and fair resolution	Art. 8, Art. 19	Art. 13	-	-	Art. 12, Art. 13

Art – article, CRC – Convention on the Rights of the Child, CRPD – Convention on the Rights of Persons with Disabilities, ICCPR – International Covenant on Civil and Political Rights, ICESCR – International Covenant on Economic, Social and Cultural Rights, UDHR – Universal Declaration of Human Rights.

### Objective 2

#### Identified principles

Among the records relevant to objective two, which outlined principles of N&FCC, seven were authored by research groups [55–61] and one by a professional association [28]. All originated from HICs. Four records were published between 2000 and 2019 [28,59–61], while the remaining four were published prior to 2000 [55–58]. Between 4 and 11 principles were reported in each reacord, yielding 57 principles in total (Table S4 in the **Online Supplementary Document**). No single record included all identified N&FCC overarching sets of principles.

#### Overarching sets of principles

**Figure 3:**
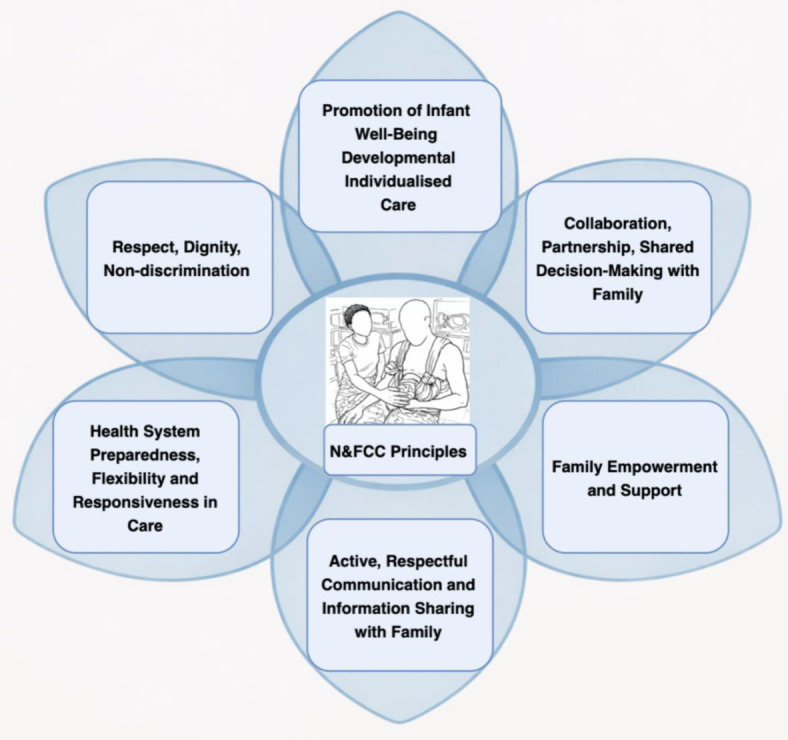
Overarching sets of N&FCC principles.

The thematic analysis of these 57 principles resulted in the identification of six overarching sets of principles for N&FCC (**Figure 3**, **Box 2**). Notably, the overarching principle ‘respect, dignity, and non-discrimination’ was articulated considering as population of interest primarily parents/family members (reported in four principles) [55,56,59,60], whereas explicit reference to the newborn was limited, with only one mention of the term ‘child’ [28]. This pattern was not observed in the analysis of the other overarching principles.

Box 2 **Overarching sets of N&FCC principles**

**Promotion of infant well-being through developmental individualised supportive care**

This principle highlights the importance of optimising infant well-being through care practices that are tailored to the individual needs, recognising non-verbal communication cues and developmental rhythms of each newborn. It recognises newborns, including those born small and sick, as active participants in their own care and emphasises the importance of providing care in a sensory environment that supports interactions conducive to optimal development.

**Collaboration, partnership, and shared decision-making with the family**

This principle emphasises meaningful collaboration between health professionals and families, including the active involvement of families, especially mothers, in care and decision-making, respect for their perspectives, and support for informed choices. Shared decision-making acknowledges parental self-determination and fosters mutual trust. Family involvement and participation are maximised when mother and infant experience zero separation, enabling delivery of key interventions such as kangaroo mother care and breastfeeding, which are critical for optimising neonatal neurodevelopmental outcomes. The principle also recognises the infant as part of a wider family system, in which other family members, particularly fathers, can support the mother–infant dyad and help create an enabling environment for care.

**Family empowerment and support**

This principle places the family, particularly key caregivers, such as the mother and the father, at the centre of the caregiving process. It emphasises the importance of empowering families through capacity building, emotional and mental health support, and active involvement in care activities. Recognising and addressing the psychosocial, emotional, and logistical burdens families face in neonatal care environments is critical to sustaining their capacity to care for their newborns in the long term and to ensure the best outcomes for newborns.

**Active, respectful communication and information sharing with the family**

This principle addresses the relational interface between the healthcare system and families. Effective, open, sensitive and respectful communication is essential to ensure families are informed and included in their newborn’s care. It reinforces the importance of building trust via transparent dialogue that is effective, appropriate and responsive to the informational needs of diverse families.

**Health system preparedness, flexibility and responsiveness in care**

This principle underscores the need for health systems to be structurally equipped and organisationally responsive to the needs of newborns, mothers and their families. This includes ensuring continuity of care, appropriate staffing, training in neonatal and family-centered practices, and flexibility in service design. Importantly, it emphasises the provision of equitable and quality care, ensuring that all families, regardless of background or circumstances, have access to high quality, appropriate, and respectful care.

**Respect – dignity – non-discrimination**

This principle emphasises the inherent value and rights of both the newborn and the family, irrespective of background, ethnicity, or socioeconomic status. It underscores the importance of recognising the newborn as a person and ensuring care is provided in a culturally sensitive and non-discriminatory way that respects newborns and their families and is attuned to infants’ and families’ individual circumstances.

#### Alignment between overarching sets of principles and overarching sets of rights

Analysis of the alignment between overarching sets of principles and overarching sets of rights showed consistent conceptual alignment, with each overarching principle aligning with one or more overarching rights. Collectively, the principles covered the full range of identified newborn rights. Similarly, all overarching sets of rights were supported across the sets of principles, with most rights reflected in multiple principles (**Table 2**), indicating a high level of interdependence between identified N&FCC principles and newborn rights.

**Table 2:** Relationship between overarching sets of principles and overarching rights*

		Overarching sets of principles					
		**Promotion of infant well-being through developmental individualised supportive care**	**Collaboration, partnership and shared decision-making with the family**	**Family empowerment and support**	**Active, respectful communication and information sharing with the family**	**Health system preparedness, flexibility, and responsiveness in care**	**Respect – dignity – non-discrimination**
**Overarching sets of rights**	**Right to freedom from harm and ill-treatment**	**✓**					
	**Right to information, informed consent, respect for choices, and supported decision-making**		**✓**		**✓**		
	**Right to privacy and confidentiality**				**✓**		
	**Right to dignity and respect**						**✓**
	**Right to personhood**	**✓**					
	**Right to equality, freedom from discrimination, and equitable care**					**✓**	**✓**
	**Right to health, healthcare, and social protection**	**✓**		**✓**		**✓**	
	**Every child has the right to be with their parents**		**✓**	**✓**			
	**Right to liberty, autonomy, self-determination and freedom from arbitrary detention**				**✓**		**✓**
	**Right to an identity and nationality from birth**	**✓**					
	**Right to adequate nutrition and clean water**	**✓**		**✓**			
	**Right to be heard and fair resolution**				**✓**		

*Ticks represent alignment between rights and principles. Absence of ticks means no alignment was identified.

## DISCUSSION

To our knowledge, this scoping review is the first to systematically identify and synthesise enunciated rights for hospitalised newborns and principles relevant to N&FCC and to examine their inter-relationships, as well as their relationship with international human rights instruments. By screening the existing literature and conducting a thematic analysis of 40 rights and 57 principles, we identified 12 overarching sets of rights specific to hospitalised newborns, each showing correspondence with at least one record in the UDHR, CRC, ICCPR, ICESCR and CRPD, and 6 overarching sets of principles for N&FCC in hospital settings which aligned with these overarching rights. These results highlight the human rights roots of N&FCC and suggest that N&FCC should be considered as a rights-based entitlement of hospitalised newborns, rather than solely as a model of care [15,35]. This is an important shift in perspective, which should foster global advocacy, promotion, and implementation of N&FCC.

Although our analysis focused on the rights of hospitalised newborns and did not explicitly examine maternal, family, or healthcare workers’ rights and needs, we note that neonatal care is inherently relational and is shaped by continuous interactions between newborns, families, and healthcare workers within an enabling health system [1,12,62]. In this context, rights are not realised in isolation; rather, the fulfilment of newborn rights is closely intertwined with the rights and needs of families and with the rights of healthcare workers to provide safe and high-quality care [15,63]. This interconnection was evident in our findings. Several newborn rights can only be meaningfully realised through family engagement, including the right to be with and cared for by the parents, and the rights to information, privacy, respectful communication and participation in decision-making, adequate nutrition and breastfeeding, which also closely overlap with family rights. In addition, mothers and family members have their own needs and rights independently from the newborn. Mothers require attentive care during the delicate postpartum period, and some need additional medical and psychosocial support related to the conditions that led to preterm birth or neonatal illness [64]. These needs of the mother-newborn dyad can be addressed through models of care that enable zero separation and place both the mother and the newborn at the centre of the care process, such as mother–newborn integrated care and couplet care. However, achieving zero separation is context dependent and can be challenging, as it often requires substantial reorganisation of care [65–67].

Following the birth of a small or sick newborn, fathers and other family members may also experience significant psychological distress and have mental health and social needs that warrant explicit attention [68–70]. Accordingly, they too may benefit from models of care that allow participation in neonatal care and interaction with the newborn.

Critically, the realisation of the right to health, healthcare and social protection, depends on respectful interactions with motivated and competent healthcare workers who are trained and adequately supported and who have the right to work in safe conditions within well-functioning and appropriate healthcare facilities [71–73], alongside policies that make healthcare accessible, affordable, equitable, and of good quality [72–77]. Accordingly, implementing N&FCC as part of a respectful and high quality care, consistent with the WHO quality of care framework, requires health systems that support not only newborns, mothers and families, but also healthcare workers through adequate staffing and resources, supportive working conditions, ongoing education, fair pay, clear roles and safe boundaries for partnership with families [14,15].

In this review, all overarching sets of principles for N&FCC aligned with overarching sets of rights, which together provide a strong conceptual foundation to guide and support N&FCC implementation. However, while recognising and protecting the roles and needs of families and healthcare workers is essential, these perspectives should be complemented by explicit attention to the newborn as the primary rights-holder and central focus of N&FCC. Our analysis of the overarching principle ‘respect, dignity, and non-discrimination’ found that explicit reference to the newborn was limited in the existing literature, highlighting an important gap: newborns are not always explicitly identified as the intended subject of N&FCC. Further research should therefore clarify the roles, scope and primary beneficiaries of N&FCC.

### Study limitations

Our thematic analysis required an interpretive analytical approach to examine the relationships between rights, international human rights instruments, and principles. Although such an analysis is inherently subjective and may be perceived as a potential source of bias, it was necessary to capture the complexity of these relationships. We minimised bias through a structured process of independent analysis conducted by three different authors, followed by discussions to resolve discrepancies.

A further limitation is the limited representation of LMIC perspectives in the identified literature. All included records articulating rights and principles were developed in HICs, with two exceptions: the WHO Patient Safety Rights Charter [43] and the White Ribbon Alliance initiative [52], both developed through multi-stakeholder collaborations across countries, including representatives from LMICs. Since our search strategy was quite broad, this imbalance may reflect reporting bias or limited inclusion of authors from LMICs in the development of existing records. Given the inductive nature of our methods, this imbalance may have contributed to an over-representation of HIC perspectives in shaping the overarching rights and principles identified. As a result, our synthesis may not fully capture how N&FCC rights and principles are understood globally. Nevertheless, the overarching rights identified were consistently aligned with international human rights instruments, which carry universal normative relevance. Future work on newborn rights and principles related to N&FCC should intentionally include stakeholders from both HICs and LMICs to better reflect diverse perspectives, strengthen contextual relevance and support implementation of N&FCC globally.

An additional limitation relates to the study selection process. Although title and abstract screening was jointly piloted on 20 records to promote consistency, the remaining records were screened primarily by one author, which may have increased the risk of selection bias. However, weekly meetings with other authors to discuss and resolve uncertainties during the screening, together with predefined eligibility criteria and multi-author review at the full-text stage, helped to mitigate this risk.

Finally, both the included literature and the perspectives informing this review were primarily rooted in medical disciplines, with limited input from non-medical fields such as law, ethics, and social sciences. Consequently, some alternative normative, legal or sociocultural interpretations of rights and principles related to N&FCC may not have been fully captured in this review, potentially affecting the breadth of out analysis.

Future research integrating multidisciplinary perspectives could strengthen the identification and articulation of newborn rights, advance the conceptual development of N&FCC and the application of human rights-based approaches, and examine how these rights can be operationalised in practice. In particular, further studies are needed to identify the governance, regulatory and accountability mechanisms required to translate newborn rights frameworks into enforceable and monitorable standards of care across different health-system contexts, including those affected by workforce shortages, infrastructure limitations and resource constraints.

Despite these limitations, this study provides an evidence synthesis that contributes to the conceptual foundations of N&FCC. By presenting these findings in a single, accessible source, this work may support clinicians, researchers, policymakers, families, and funders in promoting, implementing, and advancing N&FCC across diverse health system contexts.

## CONCLUSIONS

This scoping review is the first to systematically identify and synthesise rights for hospitalised newborns, alongside principles relevant to N&FCC, and to examine their interrelationship and correspondence with international human rights instruments. By demonstrating strong and consistent correspondence between the rights of hospitalised newborns and international human rights instruments, as well as conceptual alignment between rights and principles, our review strengthens the conceptual foundations of N&FCC and supports its implementation through a human rights-based approach. N&FCC emerges as an approach to delivering respectful, rights-based care to hospitalised newborns, their mothers and their families. Future research should clarify the roles, scope, and primary beneficiaries of N&FCC, support the development of a rights-based normative framework, examine mechanisms for translating newborn rights into enforceable standards of care, and involve stakeholders from both HICs and LMICs to support its wider implementation.

## Additional material


Online Supplementary Document
