# Supplementary material for: Newborn and family-centered care: a scoping review on related newborn rights and principles
Source: J Glob Health. 2026 Jun 26;16:04226. doi: 10.7189/jogh.16.04226 (PMC13307442; doi:10.7189/jogh.16.04226)
Supplement: Online Supplementary Document [file jogh-16-04226-s001.pdf]

Supplement to: Togo A, Lincetto O, Bua J, Mariani I, Lazzerini M. Newborn and family-centered care: a scoping review on related newborn rights and principles. J Glob Health. 2026;16:04226.

Table 1. Preferred Reporting Items for Systematic reviews and Meta-Analyses extension for Scoping Reviews (PRISMA-ScR) Checklist

SECTION	ITEM	PRISMA-ScR CHECKLIST ITEM	REPORTED ON PAGE #
**TITLE**			
Title	1	Identify the report as a scoping review.	1
**ABSTRACT**			
Structured summary	2	Provide a structured summary that includes (as applicable): background, objectives, eligibility criteria, sources of evidence, charting methods, results, and conclusions that relate to the review questions and objectives.	2
**INTRODUCTION**			
Rationale	3	Describe the rationale for the review in the context of what is already known. Explain why the review questions/objectives lend themselves to a scoping review approach.	4
Objectives	4	Provide an explicit statement of the questions and objectives being addressed with reference to their key elements (e.g., population or participants, concepts, and context) or other relevant key elements used to conceptualize the review questions and/or objectives.	4
**METHODS**			
Protocol and registration	5	Indicate whether a review protocol exists; state if and where it can be accessed (e.g., a Web address); and if available, provide registration information, including the registration number.	4
Eligibility criteria	6	Specify characteristics of the sources of evidence used as eligibility criteria (e.g., years considered, language, and publication status), and provide a rationale.	5
Information sources*	7	Describe all information sources in the search (e.g., databases with dates of coverage and contact with authors to identify additional sources), as well as the date the most recent search was executed.	5
Search	8	Present the full electronic search strategy for at least 1 database, including any limits used, such that it could be repeated.	5 and supplemental table 2
Selection of sources of evidence†	9	State the process for selecting sources of evidence (i.e., screening and eligibility) included in the scoping review.	5-6
Data charting process‡	10	Describe the methods of charting data from the included sources of evidence (e.g., calibrated forms or forms that have been tested by the team before their use, and whether data charting was done independently or in duplicate) and any processes for obtaining and confirming data from investigators.	6
Data items	11	List and define all variables for which data were sought and any assumptions and simplifications made.	6
Critical appraisal of individual sources of evidence§	12	If done, provide a rationale for conducting a critical appraisal of included sources of evidence; describe the methods used and how this information was used in any data synthesis (if appropriate).	6-7

Synthesis of results	13	Describe the methods of handling and summarizing the data that were charted.	8

KNOWLEDGE TRANSLATION PROGRAM

St. Michael's

Inspired Care.
Inspiring Science.

SECTION	ITEM	PRISMA-ScR CHECKLIST ITEM	REPORTED ON PAGE #
**RESULTS**			
Selection of sources of evidence	14	Give numbers of sources of evidence screened, assessed for eligibility, and included in the review, with reasons for exclusions at each stage, ideally using a flow diagram.	8,
Characteristics of sources of evidence	15	For each source of evidence, present characteristics for which data were charted and provide the citations.	8
Critical appraisal within sources of evidence	16	If done, present data on critical appraisal of included sources of evidence (see item 12).	8
Results of individual sources of evidence	17	For each included source of evidence, present the relevant data that were charted that relate to the review questions and objectives.	8-18
Synthesis of results	18	Summarize and/or present the charting results as they relate to the review questions and objectives.	8-18
**DISCUSSION**			
Summary of evidence	19	Summarize the main results (including an overview of concepts, themes, and types of evidence available), link to the review questions and objectives, and consider the relevance to key groups.	19
Limitations	20	Discuss the limitations of the scoping review process.	20
Conclusions	21	Provide a general interpretation of the results with respect to the review questions and objectives, as well as potential implications and/or next steps.	20
**FUNDING**			
Funding	22	Describe sources of funding for the included sources of evidence, as well as sources of funding for the scoping review. Describe the role of the funders of the scoping review.	N/A

JBI = Joanna Briggs Institute; PRISMA-ScR = Preferred Reporting Items for Systematic reviews and Meta-Analyses extension for Scoping Reviews.

* Where *sources of evidence* (see second footnote) are compiled from, such as bibliographic databases, social media platforms, and Web sites.

† A more inclusive/heterogeneous term used to account for the different types of evidence or data sources (e.g., quantitative and/or qualitative research, expert opinion, and policy documents) that may be eligible in a scoping review as opposed to only studies. This is not to be confused with *information sources* (see first footnote).

‡ The frameworks by Arksey and O'Malley (6) and Levac and colleagues (7) and the JBI guidance (4, 5) refer to the process of data extraction in a scoping review as data charting.

§ The process of systematically examining research evidence to assess its validity, results, and relevance before using it to inform a decision. This term is used for items 12 and 19 instead of "risk of bias" (which is more applicable to systematic reviews of interventions) to include and acknowledge the various sources of evidence that may be used in a scoping review (e.g., quantitative and/or qualitative research, expert opinion, and policy document).

Table 2. SEARCH Strategy Online File

PubMed/Medline/Web of Science

#1 TITLE/ABSTRACT
"Person-centered-care" OR "Person-centred-care" OR "family-centered-care" OR "family-centred-care" OR "IFCDC" OR "FCNDC" OR "family-integrated-care" OR "FIC" OR "family-participatory-care" OR "kangaroo-mother-care" OR "kangaroo-care" OR "patient-centered-care" OR "patient-centred-care" OR "KMC" OR "skin-to-skin" OR "mother-nicu-care" OR "rooming-in" OR "Mother-Neonatal-Intensive-Care" OR "Open-neonatal-unit" OR "Open-neonatal-care" OR "Open-neonatal-ward*" OR "Mother-NICU" OR "Nurturing-care" OR "People-centered-care" OR "People-centred-care" OR "NIDCAP" OR "FICARE" OR "developmental-care"
#2 TITLE/ABSTRACT
"Newborn*" OR "neonat*" OR "preterm" OR "pre-term" OR "prematur*" OR "infan*" OR "baby" OR "babies"
#3 TITLE/ABSTRACT
"principle*" OR "Concept*" OR "definition*" OR "guideline*" OR "standard*" OR "categor*" OR "implementation" OR "program*" OR "protocol*" OR "consensus" OR "clinical-practice*"OR "right*"

#4 ALL FIELDS
NOTNLM OR publisher[sb] OR inprocess[sb] OR pubmednotmedline[sb] OR indatareview[sb] OR pubstatusaheadofprint
#5 #1 AND #2 AND #3 AND #4

Google Scholar
Person centered care | Person centred care | family centered care | family centred care | IFCDC | IFCNDC | family integrated care | FIC | family participatory care | kangaroo mother care | kangaroo care | patient centered care | patient centred care | KMC | skin to skin | mother nicu care | rooming in | Mother Neonatal Intensive Care | Open neonatal unit | Open-neonatal care | Open neonatal ward* | Mother NICU | Nurturing care | People centered care | People centred care | NIDCAP | FICARE | developmental care
Newborn* | neonat* | preterm | pre-term | prematur* | infan* | baby | babies
principle* | Concept* | definition* | guideline* | standard* | categor* | implementation | program* | protocol* | consensus | clinical practice*

Newborn rights | Neonatal rights | baby rights |
Newborn* | neonat* | preterm | pre-term | prematur* | infan* | baby | babies
right* | law* | framework* | charter*

EMBASE

("Person centered care" OR "Person-centered care" OR "Person centred care" OR "Person-centred care" OR "family centered care" OR "family-centered care" OR "family centred care" OR "family-centred care" OR "FCC" OR "family-centered developmental care" OR "family centered developmental care" OR "Family-centered newborn developmental care" OR "Family centrered newborn developmental care" OR "Infant family-centered developmental care" OR "infant family centered developmental care" OR "IFCDC" OR "FCNDC" OR "family integrated care" OR "FIC" OR "family participatory care" OR "kangaroo mother care" OR "KMC" OR "skin-to-skin" OR "mother-nicu care" OR "mother nicu care" OR "rooming-in" OR "Mother Neonatal Intensive Care Unit" OR "Open neonatal unit" OR "Mother NICU" OR "Mother-NICU" OR "Nurturing care" OR "People centered care" OR "People centred care" OR "NIDCAP" OR "FICARE" OR "developmental care") AND (Newborn* OR "neonatal care" OR "small and sick newborns" OR "preterm") AND ("principle*" OR Concept* OR "definition*" OR guideline* OR standard* OR categor* OR right) AND ([embryo]/lim OR [fetus]/lim OR [infant]/lim OR [newborn]/lim)

TABLE 3 Identified rights, thematic analysis and alignment with international human rights law instruments

Overarching rights	Included articles/documents relevant to objective 1				International human rights instruments				
	White Ribbon Alliance – Respectful Maternity Care Charter 2011	World Health Organization - Patient Safety Rights 2024	NICU Baby's Bills of Rights 2024	Parma Charter on the Rights of the Newborn 2011	UDHR	CRC	International Covenant on Civil and Political Rights 1966	International Covenant on Economic, Social and Cultural Rights 1966	Convention on the rights of persons with disabilities
Right to freedom from harm and ill-treatment	Everyone has the right to freedom from harm and ill-treatment				**UDHR Art.5** No one should be subjected to torture or to cruel, inhuman or degrading treatment or punishment.	**CRC Art.19** The State shall take all the appropriate legislative, administrative, social and education measures to protect the child from all forms of physical or mental violence, injury or abuse, neglect or negligent treatment or exploitation, including sexual abuse, while in the care of parents, legal guardian or any other person who has the care of the child.	**Art 7.** No one shall be subjected to torture or to cruel, inhuman or degrading treatment or punishment. In particular, no one shall be subjected without his free consent to medical or scientific experimentation.		**Art 15.** No one shall be subjected to torture or to cruel, inhuman or degrading treatment or punishment. In particular, no one shall be subjected without his or her free consent to medical or scientific experimentation.<br><br>**Art 16** States Parties shall take all appropriate legislative, administrative, social, educational and other measures to protect persons with disabilities, both within and outside the home, from all forms of exploitation, violence and abuse, including their gender-based aspects.

Right to information, informed consent, respect for choices and preferences, supported decision-making	Everyone has the right to information, informed consent, and respect for their choices and preferences, including companion of choice during maternity care and refusal of medical procedures.	a. Right to information, education and supported decision making. b. Right to access to medical records		No medical procedure, including those for research purpose, may be performed on the newborn without the informed consent of his parents or legal guardian.			**Art 18** Everyone shall have the right to freedom of thought, conscience and religion.<br>**Art 19**<br>1. Everyone shall have the right to hold opinions without interference.<br>2. Everyone shall have the right to freedom of expression; this right shall include freedom to seek, receive and impart information and ideas of all kinds, regardless of frontiers, either orally, in writing or in print, in the form of art, or through any other media of his choice.		**Art 21.** States Parties shall take all appropriate measures to ensure that persons with disabilities can exercise the right to freedom of expression and opinion, including the freedom to seek, receive and impart information and ideas on an equal basis with others and through all forms of communication of their choice.
Right to privacy and confidentiality	Everyone has the right to privacy and confidentiality.	Right to dignity, respect, non-discrimination, privacy and confidentiality			UDHR Art.12<br>No one should be subjected to arbitrary interference with his privacy, family, home or correspondence, nor to attacks upon his honour and reputation. Everyone has the right to the protection of the law against such interference or attacks.	**CRC Art. 16**<br>1. No child shall be subjected to arbitrary or unlawful interference with his or her privacy, family, home or correspondence, nor to unlawful attacks on his or her honour and reputation.<br>2. The child has the right to the protection of the law against such	**Art 17.** No one shall be subjected to arbitrary or unlawful interference with his privacy, family, home or correspondence, nor to unlawful attacks on his honour and reputation.		**Art 22.** No person with disabilities, regardless of place of residence or living arrangements, shall be subjected to arbitrary or unlawful interference with his or her privacy, family, or correspondence or other types of communication or to unlawful attacks on his or her honour and reputation. Persons with disabilities have the right to the protection of the

						interference or attacks.			law against such interference or attacks.
Right to dignity and respect	Everyone is their own person from the moment of birth and has the right to be treated with dignity and respect.	Right to dignity, respect, non-discrimination, privacy and confidentiality		The newborn is a person and as such he is entitled to the full respect of his dignity.	UNHR Art.1 All human beings are born free and equal in dignity and rights. They are endowed with reason and conscience and should act towards one another in a spirit of brotherhood.				**Art 3.** Respect for inherent dignity, individual autonomy including the freedom to make one's own choices, and independence of persons;
Right to personhood	Everyone is their own person from the moment of birth and has the right to be treated with dignity and respect.		Right to Personhood	The newborn is a person and as such he is entitled to the full respect of his dignity.	UNHR Art.6 Everyone has the right of recognition everywhere as a person before the law.		**Art 1:** All peoples have the right of self-determination. By virtue of that right they freely determine their political status and freely pursue their economic, social and cultural development. **Art 16.** Everyone shall have the right to recognition everywhere as a		**Art 10** States Parties reaffirm that every human being has the inherent right to life and shall take all necessary measures to ensure its effective enjoyment by persons with disabilities on an equal basis with others. **Art 17** Every person with disabilities has a right to respect for his or her physical and mental

							person before the law		integrity on an equal basis with others.
Right to equality, freedom from discrimination and equitable care.	Everyone has the right to equality, freedom from discrimination and equitable care	Right to dignity, respect, non-discrimination, privacy and confidentiality			UNHR Art.2 Everyone is entitled to all the rights and freedoms set forth in this Declaration, without distinction of any kind, such as race, colour, sex, language, religion, political or other opinion, national or social origin, property, birth or other status. Furthermore, no distinction shall be made on the basis of the politica, jurisdictional or international status of the country or territory to which a person belongs, whether it be independent, trust, non-self-governing or under any other limitation of	**CRC Art. 2** State Parties shall respect and ensure the rights set forth in the present Convention to each child within their jurisdiction without discrimination of any kind, irrespective of the child's or his or her parent's/legal guardian's race, color, sex, language, religion, political or other opinion, national ethnic or social origin, property, disability, birth or other status.	**Art 2:** The States Parties to the present Covenant undertake to guarantee that the rights enunciated in the present Covenant will be exercised without discrimination of any kind as to race, colour, sex, language, religion, political or other opinion, national or social origin, property, birth or other status. **Art 3.** The States Parties to the present Covenant undertake to ensure the equal right of men and women to the enjoyment of all economic, social and cultural rights set forth in the present Covenant. **Art 26** All persons are equal before the law and are entitled without any	**Art 2:** The States Parties to the present Covenant undertake to guarantee that the rights enunciated in the present Covenant will be exercised without discrimination of any kind as to race, colour, sex, language, religion, political or other opinion, national or social origin, property, birth or other status. **Art 3.** The States Parties to the present Covenant undertake to ensure the equal right of men and women to the enjoyment of all economic, social and cultural rights set forth in the present Covenant.	**Art 5** States Parties recognize that all persons are equal before and under the law and are entitled without any discrimination to the equal protection and equal benefit of the law. States Parties shall prohibit all discrimination on the basis of disability and guarantee to persons with disabilities equal and effective legal protection against discrimination on all grounds. **Art 7** States Parties shall take all necessary measures to ensure the full enjoyment by children with disabilities of all human rights and fundamental freedoms on an equal basis with other children. In all actions concerning children with disabilities, the best interests of the child shall be a primary consideration.

					sovereignity.<br><br>UNHR Art.7<br>All are equal before the law and are entitled without discrimination to equal protection of the law. All are entitled to equal protection against any discrimination in violation of this Declaration and against any incitement to such discrimination.		discrimination to the equal protection of the law. In this respect, the law shall prohibit any discrimination and guarantee to all persons equal and effective protection against discrimination on any ground such as race, colour, sex, language, religion, political or other opinion, national or social origin, property, birth or other status.		

Right to health, health care and social protection	Everyone has the right to healthcare and to the highest attainable level of health.	a. Right to timely, effective and appropriate care. b. Right to safe health care processes and practices. c. Right to qualified and competent health workers. d. Right to safe medical products and their safe and rational use. e. Right to safe and secure health care facilities	a. Right to Neuroprotective care. b. Right to Confident and Competent Care Giving	a. Every newborn is entitled to life and the best levels of health. b. Every live newborn is entitled to appropriate assistance during delivery. c. Every newborn, be he healthy or ill, is entitled to the best care, social protection and safety available. d. Every newborn has the right to be born in the most suitable place, considering his foreseeable care requirements, especially if he suffers from or is at risk of an illness. e. In the case of the birth of a severely ill newborn (extreme prematurity, malformations or life-threatening abnormalities, etc.) appropriate treatment must be guaranteed, including	UDHR Art.25 (1) Everyone has the right to a standard of living adequate for the health and well-being of himself and of his family, including food, clothing, housing and medical care and necessary social services, and the right to security in the event of unemployment, sickness, disability, widowhood, old age or other lack of livelihood in circumstances beyond his control (2) Motherhood and childhood are entitled to special care and assistance. All children, whether born in or out of wedlock, shall enjoy the same social protection.	**CRC Art. 24.1** State Parties recognise the right of the child to the enjoyment of the highest attainable standard of health and to facilities for the treatment of illness and rehabilitation of health.State Parties shall strive tonsure that no child is deprived his or her right of access to such health care services.<br><br>**CRC Art. 3.3** State parties shall ensure that the institutions, services and facilities responsible for the care and protection of children shall conform with the standards established by competent authorities, particularly in the areas of safety, health, in the number and suitability of their staff, as well as competent supervision.		**Art 9.** The States Parties recognize the right of everyone to social security, including social insurance. **Art 10** The States Parties to the present Covenant recognize that: 1. The widest possible protection and assistance should be accorded to the family, which is the natural and fundamental group unit of society, particularly for its establishment and while it is responsible for the care and education of dependent children. Marriage must be entered into with the free consent of the intending spouses. 2. Special protection should be accorded to mothers during a reasonable period before and after childbirth. During such period working mothers should be accorded paid leave or leave with adequate social security benefits. 3. Special measures of protection and assistance should be taken on behalf of all children and young persons without any discrimination for reasons of parentage or other conditions. Children and young persons should be protected from economic and social exploitation. Their employment in	**Art 25** States Parties recognize that persons with disabilities have the right to the enjoyment of the highest attainable standard of health without discrimination on the basis of disability. States Parties shall take all appropriate measures to ensure access for persons with disabilities to health services that are gender-sensitive, including health-related rehabilitation. **Art 28** States Parties recognize the right of persons with disabilities to an adequate standard of living for themselves and their families, including adequate food, clothing and housing, and to the continuous improvement of living conditions, and shall take appropriate steps to safeguard and promote the realization of this right without discrimination on the basis of disability. States Parties recognize the right of persons with disabilities to social protection and to the enjoyment of that right without discrimination on the basis of disability, and shall take appropriate steps to safeguard and promote the realization of this right

				palliative care and pain control, avoiding both therapeutic obstinacy and the practice of euthanasia.				work harmful to their morals or health or dangerous to life or likely to hamper their normal development should be punishable by law. States should also set age limits below which the paid employment of child labour<br>should be prohibited and punishable by law. **Art 12.** 1. The States Parties to the present Covenant recognize the right of everyone to the enjoyment of the highest attainable standard of physical and mental health.<br>2. The steps to be taken by the States Parties to the present Covenant to achieve the full realization of this right shall include those necessary for: (a) The provision for the reduction of the stillbirth-rate and of infant mortality and for the healthy development of the child; (b) The improvement of all aspects of environmental and industrial hygiene;<br>(c) The prevention, treatment and control of epidemic, endemic, occupational and other diseases; (d) The creation of conditions which would assure to all medical service and medical attention in the event of sickness. **Art 15**	

								1. The States Parties to the present Covenant recognize the right of everyone: (a) To take part in cultural life; (b) To enjoy the benefits of scientific progress and its applications;<br>(c) To benefit from the protection of the moral and material interests resulting from any scientific, literary or artistic production of which he is the author. 2. The steps to be taken by the States Parties to the present Covenant to achieve the full realization of this right shall include those necessary for the conservation, the development and the diffusion of science and culture. 3. The States Parties to the present Covenant undertake to respect the freedom indispensable for scientific research and creative activity. 4. The States Parties to the present Covenant recognize the benefits to be derived from the encouragement and development of international contacts and co-operation in the scientific and cultural fields.	

Right to liberty, autonomy, self-determination and freedom from arbitrary detention	Everyone has the right to liberty, autonomy, self-determination and freedom from arbitrary detention.				**UDHR Art.9** No one shall be subjected to arbitrary arrest, detention or exile.		**Art 9**. Everyone has the right to liberty and security of person. No one shall be subjected to arbitrary arrest or detention. No one shall be deprived of his liberty except on such grounds and in accordance with such procedure as are established by law. **Art 19** 1. Everyone shall have the right to hold opinions without interference. 2. Everyone shall have the right to freedom of expression; this right shall include freedom to seek, receive and impart information and ideas of all kinds, regardless of frontiers, either orally, in writing or in print, in the form of art, or through any other media of his choice. **Art 24:** 1. Every child shall have, without any discrimination as to race, colour, sex, language, religion, national or social origin, property or birth, the right to such measures of protection as are	**Art 11**. 1. The States Parties to the present Covenant recognize the right of everyone to an adequate standard of living for himself and his family, including adequate food, clothing and housing, and to the continuous improvement of living conditions. The States Parties will take appropriate steps to ensure the realization of this right, recognizing to this effect the essential importance of international cooperation based on free consent. 2. The States Parties to the present Covenant, recognizing the fundamental right of everyone to be free from hunger, shall take, individually and through international co-operation, the measures, including specific programmes.	**Art 3** Respect for inherent dignity, individual autonomy including the freedom to make one's own choices, and independence of persons;

							required by his status as a minor, on the part of his family, society and the State.		
Every child has the right to be with their parents or guardians	Every child has the right to be with their parents or guardians	Right to patient and family engagement	a. Right to my Parents' Care. b. Right to Bond with my family. c. Right to Family Centered Care. d. Right to Healthy and Supported Parents. e. Right to Inclusion and Belonging	The newborn must be guaranteed vicinity to his parents.		**CRC Art.9** (1) States Parties shall ensure that a child shall not be separated from his or her parents against their will, except when competent authorities subject to judicial review determine, in accordance with applicable law and procedures, that such separation is necessary for the best interests of the child. Such determination may be necessary in a particular case such as one involving abuse or neglect of the child by the parents, or one where the parents are living separately and a decision must be made as to the child's place of residence.	**Art 23** 1. The family is the natural and fundamental group unit of society and is entitled to protection by society and the State.		**Art 23.** States Parties shall ensure that a child shall not be separated from his or her parents against their will.
Right to an identity and nationality from birth.	Every child has the right to an identity and nationality from birth.			Every newborn is entitled to be registered after birth, to be given a name and to acquire a nationality.		**CRC Art.7** (1) The child shall be registered immediately after birth and shall have the right from birth to a name, the right to acquire a nationality and. as	**Art 24.** 2. Every child shall be registered immediately after birth and shall have a name. 3. Every child has the right to acquire a nationality.		**Art 18.** States Parties shall recognize the rights of persons with disabilities to liberty of movement, to freedom to choose their residence and to a nationality, on an equal basis with others, including by

						far as possible, the right to know and be cared for by his or her parents.			ensuring that persons with disabilities:
Right to adequate nutrition and clean water.	Everyone has the right to adequate nutrition and clean water.		Right to be Nourished	Every newborn is entitled to be adequately fed, to guarantee his best psychological and physical development.	UDHR Art.25.<br>(1) Everyone has the right to a standard of living adequate for the health and well-being of himself and of his family, including food, clothing, housing and medical care and necessary social services, and the right to security in the event of unemployment, sickness, disability, widowhood, old age or other lack of livelihood in circumstances beyond his control<br>(2) Motherhood and childhood are entitled to special care and assistance. All children, whether born in or out of wedlock, shall enjoy the same social protection.	**CRC Art.24**<br><br>(e) To ensure that all segments of society, in particular parents and children, are informed, have access to education and are supported in the use of basic knowledge of child health and nutrition, the advantages of breastfeeding, hygiene and environmental sanitation and the prevention of accidents;			**Art 28** States Parties recognize the right of persons with disabilities to an adequate standard of living for themselves and their families, including adequate food, clothing and housing, and to the continuous improvement of living conditions, and shall take appropriate steps to safeguard and promote the realization of this right without discrimination on the basis of disability.

Right to be heard and fair resolution		Right to be heard and fair resolution	Right to Advocacy		UDHR Art.19<br>Everyone has the right to freedom of opinion and expression; this right includes freedom to hold opinions without interference and to seek, receive and impart information and ideas through any media and regardless of frontiers.<br><br>UDHR Art.8<br>Everyone has the right to an effective remedy by the competent national tribunals for acts violating the fundamental rights granted him by the constitution or by law.	**CRC Art.13**<br>1. The child shall have the right to freedom of expression; this right shall include freedom to seek, receive and impart information and ideas of all kinds, regardless of frontiers, either orally, in writing or in print, in the form of art, or through any other media of the child's choice.			**Art 12.** States Parties reaffirm that persons with disabilities have the right to recognition everywhere as persons before the law.<br><br>**Art 13** States Parties shall ensure effective access to justice for persons with disabilities on an equal basis with others, including through the provision of procedural and age-appropriate accommodations, in order to facilitate their effective role as direct and indirect participants, including as witnesses, in all legal proceedings, including at investigative and other preliminary stages.

TABLE 4. Synthesis of overarching sets of principles from thematic analysis and alignment with overarching sets of rights

	Principles (source at the bottom of the row)	Overarching sets of principles derived from thematic analysis of principles
1.	Listening to and respecting each child and his/her family. Honouring racial, ethnic, cultural, and socioeconomic background and patient/family experiences and incorporating them in accordance with patient/family preference into the planning and delivery of health care.<br><br>(Committee On Hospital, Institute For et al. 2012)	a. Respect – Dignity – Non-Discrimination<br><br>f. Health System Preparedness, Flexibility and Responsiveness in Care
2.	Ensuring flexibility in organizational policies, procedures and provider practices so services can be tailored to the needs, beliefs, and cultural values of each child/family and facilitating choice for the child/family about approaches to care.<br><br>(Committee On Hospital, Institute For et al. 2012)	d. Collaboration and Partnership and Shared Decision-Making<br><br>f. Health System Preparedness, Flexibility and Responsiveness in Care
3.	Sharing complete, honest, and unbiased information with patients and their families on an ongoing basis and in ways they find useful and affirming, so that they may effectively participate in care and decision-making to the level they choose. Health information for children/families should be available in the range of cultural and linguistic diversity in the community and take into account health literacy.<br><br>(Committee On Hospital, Institute For et al. 2012)	c. Active Respectful Communication and Information Sharing with the Family<br><br>d. Collaboration and Partnership and Shared Decision-Making

4. Providing and/or ensuring formal and informal support for the child and family during each phase of the child's life, following ethical and legal guidelines.<br><br>(Committee On Hospital, Institute For et al. 2012)	e. Parental Empowerment and Support<br><br>b. Infant Well-Being and Developmental Individualised Supportive Care
5. Collaborating with patients and families at all levels of health care: in the delivery of care to the individual child, in professional education, policy making, program development, implementation, and evaluation, and in health care facility design.<br><br>(Committee On Hospital, Institute For et al. 2012)	**d.** Collaboration and Partnership and Shared Decision-Making<br><br>**f.** Health System Preparedness, Flexibility and Responsiveness in Care
6. Recognizing and building on the strengths of individual children and families and empowering them to discover their own strengths, build confidence and participate in making choices and decisions about their health care.<br><br>(Committee On Hospital, Institute For et al. 2012)	**b.** Infant Well-Being and Developmental Individualised Supportive Care<br><br>**e.** Parental Empowerment and Support<br><br>**d.** Collaboration and Partnership and Shared Decision-Making

7. Recognition that the family is the constant in the child's life while the service system and personnel within those systems fluctuate.<br><br>(Korteland and Cornwell 1991)	**e.** Parental Empowerment and Support
8. Facilitation of parent/professional collaboration.<br><br>(Korteland and Cornwell 1991)	**d.** Collaboration and Partnership and Shared Decision-Making
9. Sharing of unbiased and complete information with parents about their child's care on an ongoing basis in an appropriate and supportive manner.<br><br>(Korteland and Cornwell 1991)	**c.** Active Respectful Communication and Information Sharing with the Family
11. Implementation of appropriate policies in programs that are comprehensive and provide emotional and financial support to meet the needs of families.<br><br>(Korteland and Cornwell 1991)	**e.** Parental Empowerment and Support<br><br>**f.** Health System Preparedness, Flexibility and Responsiveness in Care
12. Recognition of family strengths and individualities and respect for different methods of coping.<br><br>(Korteland and Cornwell 1991)	**a.** Respect – Dignity – Non-Discrimination<br><br>**e.** Parental Empowerment and Support

12. Understanding and incorporating the developmental needs of infants, children and adolescents and their families into health care delivery systems.<br><br>(Korteland and Cornwell 1991)	**b.** Infant Well-Being and Developmental Individualised Supportive Care<br><br>**f.** Health System Preparedness, Flexibility and Responsiveness in Care
13. Encouragement and facilitation of parent-to-parent support.<br><br>(Korteland and Cornwell 1991)	**e.** Parental Empowerment and Support
14. Assurance that the design of health care delivery systems is flexible, accessible and responsive to family needs.<br><br>(Korteland and Cornwell 1991)	**d.** Collaboration and Partnership and Shared Decision-Making<br><br>**f.** Health System Preparedness, Flexibility and Responsiveness in Care
15. Honouring of racial, ethnic, cultural and socioeconomic diversity of families.<br><br>(Korteland and Cornwell 1991)	**a.** Respect – Dignity – Non-Discrimination
16. Treat parents with dignity and respect and develop a relationship based on trust.<br><br>Staniszewska S, et al 2012	**a.** Respect – Dignity – Non-Discrimination<br><br>**d.** Collaboration and Partnership and Shared Decision-Making

17. Involve parents in all aspects of their infant's care.<br><br>Staniszewska S, et al 2012	**d.** Collaboration and Partnership and Shared Decision-Making<br><br>**e.** Parental Empowerment and Support
18. Be aware of parents' needs, the emotional impact of preterm birth and individual differences in parental responses and needs. Recognize critical steps for parents on the care pathway<br><br>Staniszewska S, et al 2012	**e.** Parental Empowerment and Support
19. Recognise and value the roles of parents, siblings and other family members<br><br>Staniszewska S, et al 2012	**e.** Parental Empowerment and Support
20. Provide sensitive and compassionate interaction.<br><br>Staniszewska S, et al 2012.	**c.** Active Respectful Communication and Information Sharing with the Family
21. Provide practical help with infant care and parent interaction, including identification of behavioural cues.<br><br>Staniszewska S, et al 2012	**b.** Infant Well-Being and Developmental Individualised Supportive Care<br><br>**e.** Parental Empowerment and Support
22. Provide psychosocial support for parents.<br><br>Staniszewska S, et al 2012	**e.** Parental Empowerment and Support

23. Promote parenting skills in caring for an interacting with their infant.<br><br>Staniszewska S, et al 2012	**b.** Infant Well-Being and Developmental Individualised Supportive Care<br><br>**e.** Parental Empowerment and Support
24. Maximise opportunities for communication with parents and parent groups.<br><br>Staniszewska S, et al 2012	**c.** Active Respectful Communication and Information Sharing with the Family<br><br>**d.** Collaboration and Partnership and Shared Decision-Making
25. Support mothers' ability to nurture their baby by expressing breast milk and breastfeeding.<br><br>Staniszewska S, et al 2012	**b.** Infant Well-Being and Developmental Individualised Supportive Care<br><br>**e.** Parental Empowerment and Support
26. Provide appropriate family-friendly facilities.<br><br>Staniszewska S, et al 2012	**e.** Parental Empowerment and Support
27. People are treated with dignity and respect<br><br>Dunn M.S., et al 2006	**a.** Respect – Dignity – Non-Discrimination

28. Health care providers communicate and share complete and unbiased information with patients and families in ways that are affirming and useful<br><br>Dunn M.S., et al 2006	**c.** Active Respectful Communication and Information Sharing with the Family
29. Patients and family members build on their strengths by participating in experiences that enhance control and independence<br><br>Dunn M.S., et al 2006	**e.** Parental Empowerment and Support
30. Collaboration among patients, family members, and providers occurs in policy and program development, professional education, and the delivery of care<br><br>Dunn M.S., et al 2006	**d.** Collaboration and Partnership and Shared Decision-Making<br>**f.** Health System Preparedness, Flexibility and Responsiveness in Care
31. Recognizing the family as a constant in the child's life<br><br>Shelton T.L., et al (1987)	**e.** Parental Empowerment and Support
32. Facilitating parent-professional collaboration at all levels of healthcare<br><br>Shelton T.L., et al (1987)	**d.** Collaboration and Partnership and Shared Decision-Making
33. Honouring the racial, ethnic, cultural, and socio-economic diversity of families<br><br>Shelton T.L., et al (1987)	**a.** Respect – Dignity – Non-Discrimination

34. Recognizing family strengths and individuality and respecting different methods of coping<br><br>Shelton T.L., et al (1987)	**a.** Respect – Dignity – Non-Discrimination
35. Sharing complete and unbiased information with families on a continuous basis<br><br>Shelton T.L., et al (1987)	**c.** Active Respectful Communication and Information Sharing with the Family
36. Encouraging and facilitating family to family support and networking<br><br>Shelton T.L., et al (1987)	**e.** Parental Empowerment and Support
37. Responding to child and family developmental needs as part of health care practices<br><br>Shelton T.L., et al (1987)	**b.** Infant Well-Being and Developmental Individualised Supportive Care
38. Adopting policies and practices that provide families with emotional and financial support<br><br>Shelton T.L., et al (1987)	**e.** Parental Empowerment and Support
39. Designing health care that is flexible, culturally competent, and responsive to family needs<br><br>Shelton T.L., et al (1987)	**f.** Health System Preparedness, Flexibility and Responsiveness in Care
40. Family-centered neonatal care should be based on open and honest communication between parents and professionals on medical and ethical issues<br><br>Harrison H, 1993;	**c.** Active Respectful Communication and Information Sharing with the Family

41. To work with professionals in making informed treatment choices, parents must have available to them the same facts and interpretation of those facts as the professionals, including medical information presented in meaningful formats, information about uncertainties surrounding treatments, information from parents whose children have been in similar medical situations, and access to the chart and rounds discussions<br><br>Harrison H, 1993	**c.** Active Respectful Communication and Information Sharing with the Family
42. In medical situations involving very high mortality and morbidity, great suffering, and/or significant medical controversy, fully informed parents should have the right to make decisions regarding aggressive treatment for their infants.<br><br>Harrison H, 1993	**d.** Collaboration and Partnership and Shared Decision-Making<br><br>**c.** Active Respectful Communication and Information Sharing with the Family

43. Expectant parents should be offered information about adverse pregnancy outcomes and be given the opportunity to state in advance their treatment preferences if their baby is born extremely prematurely and/or critically ill.<br><br>Harrison H, 1993	**d.** Collaboration and Partnership and Shared Decision-Making<br><br>**c.** Active Respectful Communication and Information Sharing with the Family
44. Parents and professionals must work together to acknowledge and alleviate the pain of infants in intensive care<br><br>Harrison H, 1993	**b.** Infant Well-Being and Developmental Individualised Supportive Care
45. Parents and professionals must work together to ensure an appropriate environment for babies in the neonatal intensive care<br><br>Harrison H, 1993	**b.** Infant Well-Being and Developmental Individualised Supportive Care
47. Parents and professionals must work together to develop nursery policies and programs that promote parenting skills and encourage maximum involvement of families with their hospitalized infant<br><br>Harrison H, 1993	**d.** Collaboration and Partnership and Shared Decision-Making<br><br>**e.** Parental Empowerment and Support

48. Parents and professionals must work together to promote meaningful long-term follow-up for all high-risk NICU survivors<br><br>Harrison H, 1993	**b.** Infant Well-Being and Developmental Individualised Supportive Care
49. Parents and professionals must acknowledge that critically ill newborns can be harmed by overtreatment as well as by undertreatment, and we must insist that our laws and treatment policies be based on compassion. We must work together to promote awareness of the needs of NICU survivors with disabilities to ensure adequate support for them and their families. We must work together to decrease disability through universal prenatal care<br><br>Harrison H, 1993	**b.** Infant Well-Being and Developmental Individualised Supportive Care
50. Sensitive care based on infant behavioural communication and cues gives the infant a voice<br><br>Bergman NJ, et al 2019	**b.** Infant Well-Being and Developmental Individualised Supportive Care
51. It is beneficial for brain growth<br><br>Bergman NJ, et al 2019	**b.** Infant Well-Being and Developmental Individualised Supportive Care
52. Parents engagement supports parental well-being and infant development<br><br>Bergman NJ, et al 2019	**b.** Infant Well-Being and Developmental Individualised Supportive Care<br><br>**e.** Parental Empowerment and Support
53. Customized adaptations of the NICU environment and hospital system as a whole.<br><br>Bergman NJ, et al 2019	**f**. Health System Preparedness, Flexibility and Responsiveness in Care

54. Establish and maintain adaptive fit within the family and between the family system and the service delivery system<br><br>Thurman, S. K. 1991	**d**. Collaboration and Partnership and Shared Decision-Making<br><br>**f.** Health System Preparedness, Flexibility and Responsiveness in Care
55. Provide services based on family identified needs and desires<br><br>Thurman, S. K. 1991	**f.** Health System Preparedness, Flexibility and Responsiveness in Care
56. Foster family independence and empowerment while providing a stable ongoing support system<br><br>Thurman, S. K. 1991	**e.** Parental Empowerment and Support
57. Recognize that families are complex, dynamic and ever-changing systems<br><br>Thurman, S. K. 1991	**e.** Parental Empowerment and Support

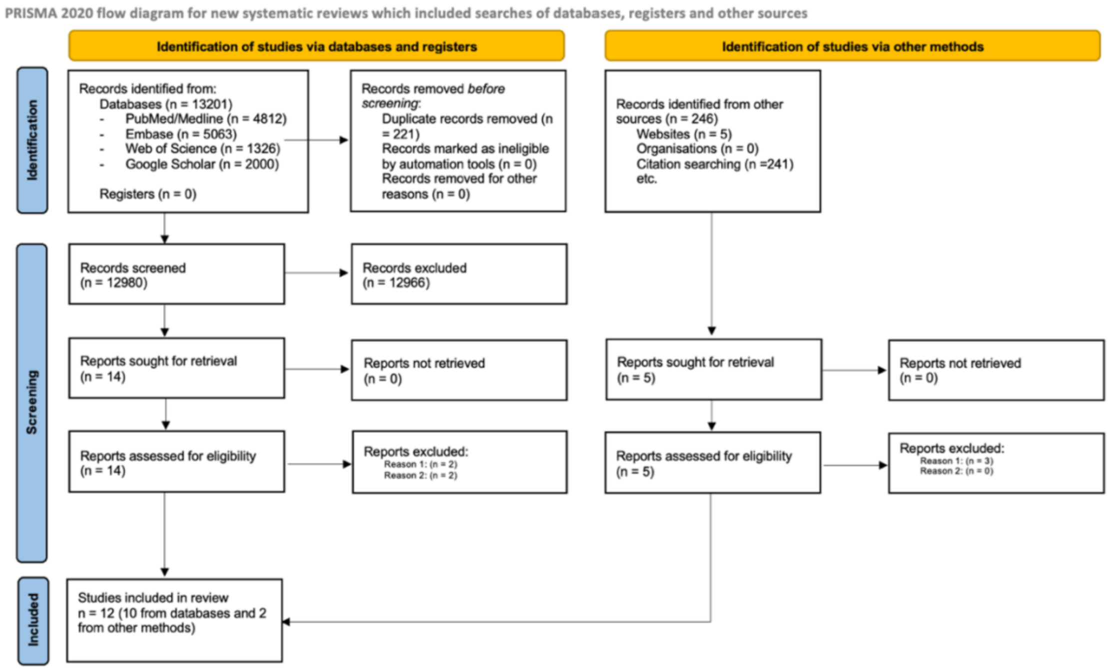

Source: Page MJ, et al. BMJ 2021;372:n71. doi: 10.1136/bmj.n71.

This work is licensed under CC BY 4.0. To view a copy of this license, visit https://creativecommons.org/licenses/by/4.0/

Figure S1. PRISMA flowchart, adapted from Page *et al*. [40]. Reason 1: records which did not specifically address the rights of hospitalised newborns or principles of N&FCC. Reason 2: language not spoken by authors (*e.g.* Chinese).
